# Essential health services delivery in South Africa during COVID-19: Community and healthcare worker perspectives

**DOI:** 10.3389/fpubh.2022.992481

**Published:** 2022-12-08

**Authors:** Samanta T. Lalla-Edward, Atiya Mosam, Jennifer Hove, Agnes Erzse, Teurai Rwafa-Ponela, Jessica Price, Athini Nyatela, Sizwe Nqakala, Kathleen Kahn, Stephen Tollman, Karen Hofman, Susan Goldstein

**Affiliations:** ^1^Ezintsha, Faculty of Health Sciences, University of the Witwatersrand, Johannesburg, South Africa; ^2^South African Medical Research Council (SAMRC)/Wits Centre for Health Economics and Decision Science – PRICELESS South Africa (SA), School of Public Health, Faculty of Health Sciences, University of the Witwatersrand, Johannesburg, South Africa; ^3^Medical Research Council (MRC)/Wits Rural Public and Health Transitions Research Unit (Agincourt), School of Public Health, Faculty of Health Sciences, University of the Witwatersrand, Johannesburg, South Africa

**Keywords:** coronavirus, healthcare, pandemic, SARS-CoV-2, treatment interruption, qualitative, lived experience, stakeholder engagement

## Abstract

**Background:**

Between May 2020 and February 2022, South Africa's health system bore strain as it battled mitigating the coronavirus pandemic. The country's pandemic response was scrutinized. This period also brought into focus pre-existing shortcomings in the healthcare system and its governing bodies. Contextually, there is a paucity in literature on the experiences of healthcare providers and users. This study aimed to contribute information on COVID-19, with the intention of providing guidance on preparing for future infectious disease outbreaks.

**Methods:**

Cross sectional exploratory qualitative methodology was employed using semi-structured interviews and focus group discussions with community members (CM) and healthcare workers (HCW) from two South African study sites: (a) rural Bushbuckridge (run by Agincourt Health and Socio-Demographic Surveillance Site) and (b), Regions D and F in Johannesburg Metropole.

**Results:**

After interviewing 42 CMs and 43 HCWs, it emerged that mandated process changes while minimizing COVID-19 exposure, necessitated healthcare personnel focusing on critical care treatment at the expense of less acute ones. COVID-19 isolation protocols, extensive absenteeism and HCWs with advanced skills being perceived as more adept to treat COVID-19 patients contributed to HCWs experiencing higher workloads. Fears regarding contracting and transmitting COVID-19, suffering financial losses, and not being able to provide adequate advice to patients were recurrent themes. Dissemination of relevant information among healthcare facility personnel and communities suffered due to breakdowns in communication.

**Conclusion:**

Concessions and novel strategies to avail medication to patients had to be created. Since providence was lacking, government needs to formulate health intervention strategies that embrace health literacy, alternate methods of chronic medication dispensation, improved communication across health care platforms and the use of telehealth, to circumvent the threats of possible further infectious disease outbreaks.

## Introduction

Ten days after identifying its first severe acute respiratory syndrome coronavirus 2 (SARS-CoV-2) case on 5 March 2020, South Africa declared a national state of disaster to address the coronavirus disease-19 (COVID-19) pandemic. Lockdowns which imposed various levels of restriction in movement and activity on the population were enforced (1). The strictest 5 week “hard lockdown” occurred from March to April 2020, where people were only permitted movement beyond the boundaries of their homes to seek emergency medical attention and to access essential services. From 3 May 2020 up until the end of February 2022, 296,224 more deaths (many from obesity related conditions) than usual were reported, 16% of which were likely due to overwhelmed health services (2, 3).

Not only has the pandemic profoundly impacted South Africa's health system, but it also exposed and deepened pre-pandemic challenges in health service access and delivery. Post-apartheid gains regarding access and quality of healthcare services have been unequal (4) with persisting disparities between socioeconomic classes, geographical locations and public vs. private health sectors (5, 6). The causes include insufficient budget, and unequal distribution of resources, especially in rural areas (6). Provinces with the largest rural population have the lowest percentage of facilities with ideal clinic status (a measure of quality primary healthcare in South Africa) (6).

Using the World Health Organization (WHO) Universal Health Coverage (UHC) index and routinely collected data it has surfaced that service coverage increased from 46.1 to 58.3 between 2007/8 and 2016/7 (7). Health worker coverage is low for the whole country. Program areas in which the poorer districts performed noticeably badly were infectious diseases, non-communicable diseases (NCD) and mother and child health (MCH) (7).

The additional burden caused by COVID-19, raised concerns about disruptions to essential health services delivery, catalyzed by the pandemic (8–10). Early model-based studies from low- and middle-income countries (LMICs) estimated that the reductions in healthcare access could cause monthly increases of 9–45% in deaths of children under-5 and 8–39% in maternal deaths (11). Furthermore, estimate predictions suggest increasing deaths due to HIV of up to 10%, and due to tuberculosis of up to 20% over 5 years, when compared with mortality estimations should the COVID-19 pandemic have not occurred (12). Some South African regions reported an 11% increase in uncontrolled diabetes and ~60% reduction in the number of hemoglobin 1AC tests being conducted (13).

Emerging research documented the observed changes in service utilization and delivery during the pandemic. Following the introduction of a national lockdown in March 2020, across almost all districts there was a substantial and enduring reduction in primary healthcare utilization during April and May 2020 (14). Services impacted most were HIV testing and health visits by children under 5 years of age, irrespective of the actual district-level incidence risk of COVID-19. Immunization and contraception services remained sub-standard in 75% of districts in August 2020. These deficiencies are corroborated by self-reported survey data from the uninsured segment of the population, where 23% were neither seeking acute care when needed nor accessing medication, contraceptives, or condoms (15). The literature attributed a large share of unmet healthcare needs to the unintended social and economic consequences of COVID-19 and the response to the pandemic (14, 16).

The response measures stopped all but essential services, reduced mobility and income and were associated with lasting impacts on unemployment, food insecurity, poverty, and public health. They also affected people's ability to access services. Barriers to accessing healthcare were worsened for individuals who were more susceptible to the economic difficulties associated with the pandemic (17).

Insights into the severity of these disruptions based on the lived experience of individuals could be important to mitigate the impact on health outcomes as the pandemic continues Literature on the lived experience of both health service users and providers is limited. Discerning the impact of COVID-19 by exploring lived realities enables us to provide context into which policies play out in practice. This evidence has the unique ability to inform strategies to: (a) maintain health services during potential future COVID-19 pandemic waves and (b) improve preparedness for future infectious disease outbreaks by enhancing health system resilience, so as not to undermine efforts already made toward UHC.

This analysis examined how individuals who seek, and receive, deliver, and manage healthcare have perceived and experienced service delivery for selected non-COVID-19 conditions before and during the pandemic. It will provide a deeper understanding of the impact of COVID-19 on health service delivery and utilization.

## Materials and methods

**Figure 1:**
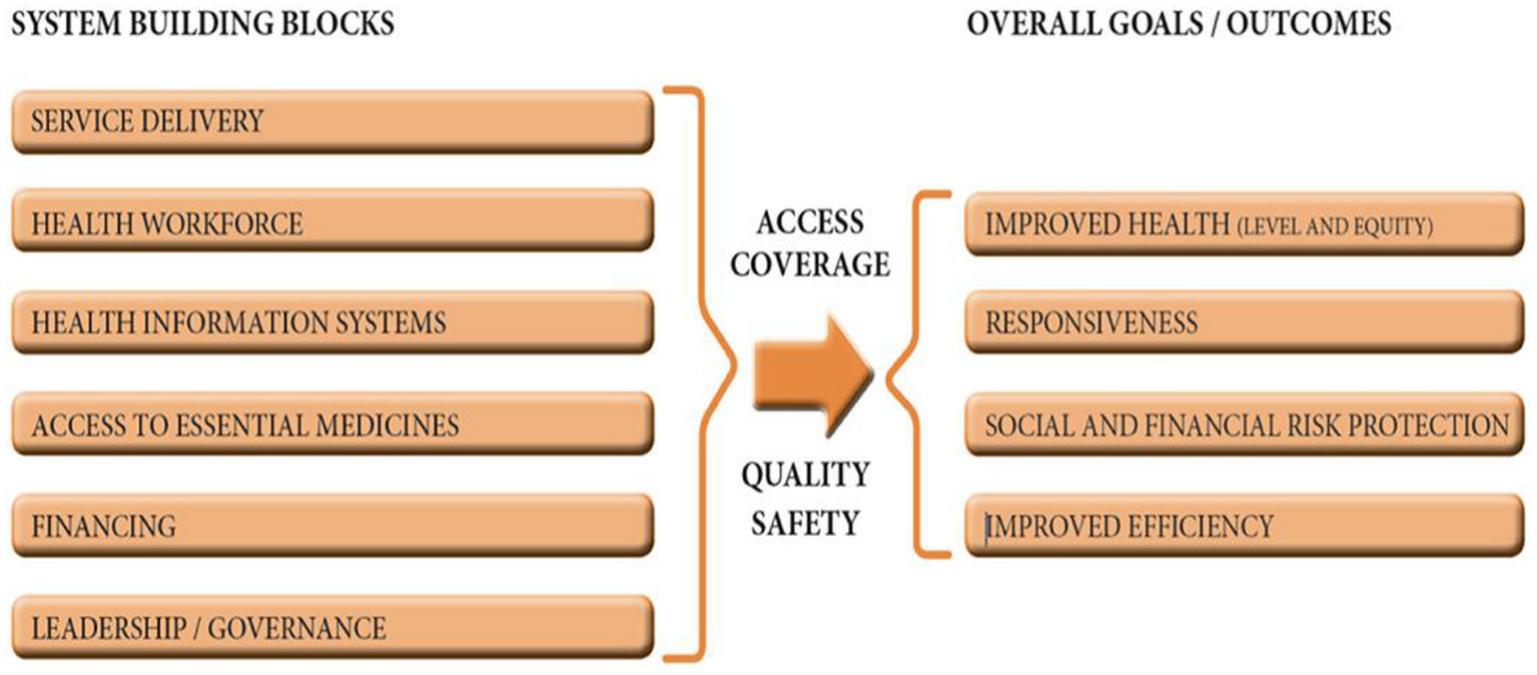
The six building blocks of a health system (18).

The achievement of UHC is dependent on a strong and resilient health care system with high-caliber essential health services. The WHO established a framework for action on strengthening health systems based on six health system building blocks (Figure 1) (18). Using thematic analysis (19), this qualitative study was conducted to report how each block was affected by the COVID-19 measures from the perspective of healthcare workers (HCW) and community members (CM). We used the Lincoln and Guba criteria (credibility, confirmability, dependability, and transferability) to guide the rigor of our data processes (20). To ensure transferability and dependability a detailed study protocol with comprehensive process documents, minutes of stakeholder engagements, and field and reflexive notes from multiple research team members have been maintained for auditing and/or data accessibility to other researchers.

### Study setting

The study was undertaken at two sites:

- The Agincourt Health and Socio-Demographic Surveillance Site (HDSS) comprising 7 primary care clinics and 2 community health centers within the study site, plus 3 district hospitals 25–60 km away, in rural Bushbuckridge, Mpumalanga, and
- Regions D and F, in inner city, urban Johannesburg. In Region D, data were collected from participants linked to two community health centers and 6 primary health clinics.

Healthcare service are primarily nurse led and include acute and chronic care, services for HIV, tuberculosis, sexual and reproductive health, maternal and child health and mobile services for some facilities.

### Study population and sample

Using a combination of our experience and guidance from Hennink et al. (21) we expected to collect data from up to 25 community members and 25 healthcare workers from each of the settings (rural and urban), i.e., ~100 participants in total until data saturation would be reached. Purposive sampling was used to identify CMs 18 years or older accessing services for HIV, non-communicable diseases, or maternal and child health, and HCWs across both sites. In Agincourt, CMs were identified through a clinic-link system which provides details on participants' diagnosis, chronic medication, utilization of services and missed visits during the COVID-19 pandemic. In Johannesburg, HCWs and health promoters worked with the study team to recruit CMs attending health facilities based on the same criteria. CMs were consented to access their healthcare records to confirm eligibility. HCWs were purposively selected based on their role, facility level and department.

### Data collection

This was a qualitative study based on in-depth interviews (IDIs) and focus group discussions (FGD). Due to COVID-19 restrictions a hybrid (telephonic and in-person) model of data collection was used. For in-person interviews COVID-19 protocols were maintained. This included mask wearing by both interviewer and participants, maintaining a social distance, interviews being conducted outdoors and sanitizing of any shared items or surfaces (e.g., pens, chairs, and tables).

IDIs and FGDs were conducted in the local languages (IsiZulu, IsiXhosa, SeSotho, SePedi, SeTswana in Johannesburg; Xitsonga in Mpumalanga) and English. Using semi-structured data collection guides, IDIs and FGDs were performed by four research experienced, protocol trained local field workers (2 males and 2 females) who are familiar with the study contexts. Data collection guides were piloted in Region F and adapted for data collection in the other research sites. Researchers performed quality checks in real time by listening to interview recordings and reading transcripts to ensure that questions were asked and interpreted as intended and that the transcripts produced were an accurate reflection of the interviews. These activities contributed to the study credibility and dependability. All data were collected between March and September 2021. Interviews lasted 35–60 min. As we were interested in community members and healthcare workers' experiences and perceptions of healthcare service delivery and utilization during COVID-19, the data collection guides focused on the following three issues: (1); perceptions and individual experience of COVID-19 and lockdown, (2) access to health services before and during COVID-19, and (3) delivery of healthcare services before and during COVID-19.

### Data analysis

All interviews were audio-recorded, transcribed, and translated verbatim into English. Transcripts were de-identified and quality checks for accuracy and completeness were conducted by the research team. The thematic analysis approach was used (19), based on Strauss and Corbin's method of open, axial and selective coding (22). To develop an initial list of codes (open coding), seven researchers independently coded three transcripts line by line, with one transcript from each of the following groups: CM individual IDIs, HCW individual IDIs and CM FGDs. A process of continuous comparison was employed whereby subsequent transcripts were coded using this is and new themes which emerged from the new transcripts were added to the list after consultation and agreement across the analysis team. Data analysis was facilitated using MAXQDA 2020 software to manage transcripts, themes and quotes. Codes were organized and re-organized into broader categories based on thematic similarities between codes (axial coding). Thereafter selective coding was conducted to place codes into categories. This was guided by a deductive approach where categories were aligned to the research question of how the WHO building blocks (18) were affected by COVID-19 measures. Eight deductive themes included: service delivery, service utilization, information and communication, health workforce, medication and resources, financial impact, quality of care, and recommendations. To ensure credibility and consistency of coding and consensus on axial and selective codes each transcript was double coded and checked by a third coder. Any discrepancies were resolved through discussions during the coding team bi-weekly meetings. To further strengthen all four aspects of trustworthiness, there were ongoing stakeholder (other researchers (monthly), field staff (bi-weekly), advisory committee (quarterly) engagements to discuss the data and findings. During these engagements the qualitative findings were presented and reviewed alongside the quantitative service delivery results (published elsewhere) to assess confirmability.

### Ethical considerations

Ethical clearance for the overall study was received from the University of the Witwatersrand Human Research Ethics Committee (M201084) in January 2021. Departmental approval was granted in February 2021 by the Johannesburg Health District (DRC Ref: 2020-11-015), Mpumalanga Province Ethics Committee (MP_202102_03) and National Health Research Database (GP_202011_066). All participants provided informed consent for study participation and recording prior to data collection commencement; community members received ZAR 300 (approximately USD 17.60) reimbursement for their time and travel in Johannesburg, whereas interviews were conducted at respondent's home in the Agincourt HDSS.

## Results

**Figure 2:**
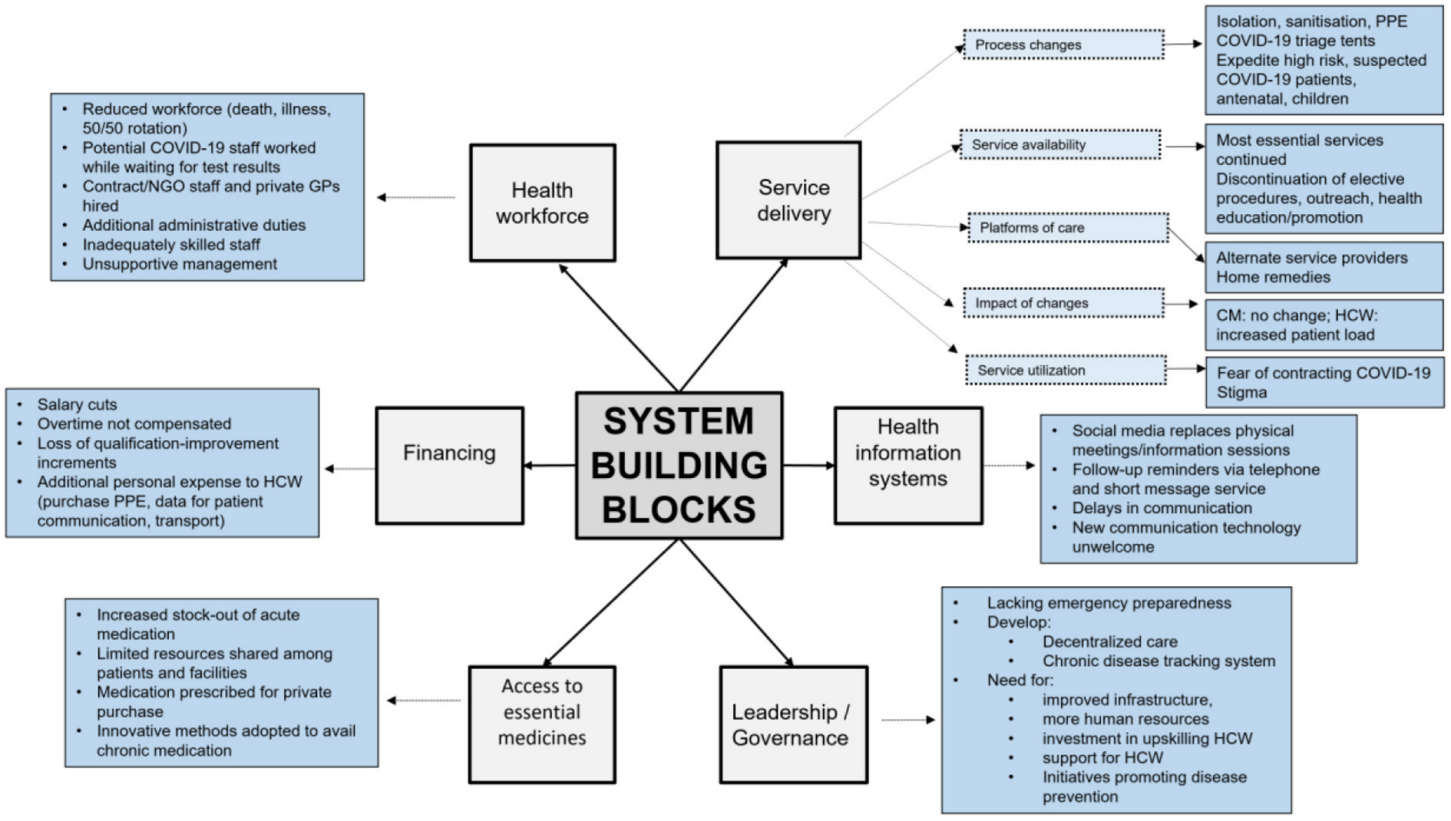
Summary of the thematic analysis.

Section Introduction of the results gives an overview of participant demographics. Section Materials and methods presents the qualitative results using the following categories: service delivery, service utilization, information and communication, health workforce, medication and resources, financing, quality of care, and recommendations for government (summarized in Figure 2). The results are presented in accordance with consolidated criteria for reporting qualitative research (COREQ) guidelines (23).

### Participant demographics

Forty-three HCWs and forty-two CMs participated in the study. There were more HCWs from Johannesburg (JHB), and slightly more CMs from Agincourt (AGIN). Over 75% of participants were women, with no men participants in the CM FGD group in either site. Clinics yielded most HCW participants in both sites. Treatment services for hypertension and HIV were the most accessed services in the CM IDI group in Agincourt (Table 1).

**Table 1:** Participant demographics.

		**Agincourt**	**Johannesburg**
**HCWs included: operational managers, doctors, clinical associates, nurses and community health workers from clinics, community health centers and district hospitals**.			
Total		18	25
Gender	Women	11	19
	Men	7	6
**Community members included people who accessed chronic services for illnesses such as hypertension, diabetes, HIV infection, as well as those accessing mother and child health services**.			
Total		24	17
Gender	Women	19	14
	Men	5	3
Service accessed^*^	Chronic (Diabetes)	4	3
	Chronic (Hypertension)	9	6
	Chronic (HIV)	14	4
	Maternal health	1	7
	Child health	5	8
	Other	3	4
**Two community member focus group discussions**			
Total		4	6
Gender	Women	4	6
	Men	0	0

HCW, healthcare worker; CHW, community health worker; CHC, community health center; IDI, in-depth interview; FGD, focus group discussion.

* CMs may have accessed more than one service.

### Thematic analysis

Overall, participants believed that COVID-19 did not worsen service delivery but rather exposed, “*the insufficiencies in the system… (when) we needed the health system to be more robust now.”* (JHB HCW) Despite this, HCWs stated that COVID-19, “*also exposed it in a good way…. a lot of people have a bad perception of our public healthcare and they saw that we are managing with the patients, and we are doing quite well.”* (JHB HCW)

#### Service delivery

##### Process changes

Implemented process changes included screening and triage at the facility gates, outside waiting areas, queue cut-offs, booking systems for chronic patients, batching of patients being allowed into the facility buildings and prohibiting patient escorts into the facility. Additionally, HCWs and CMs reported that high-risk patients, suspected COVID-19 cases, antenatal patients, and children were expedited through the facility. Where possible, services were integrated to ensure expedient patient care, e.g., antenatal and HIV. A JHB HCW explained the frustrations resulting from the inability to deliver holistic care: “*And with chronic patients, if they are only here for medication, we don't even ask them ‘would you want to test today…', we just give … whatever that they are here for and then they leave…we couldn't manage patients holistically, it affected us a lot”*.

HCWs discussed infection control processes (which included sanitization of the facility and equipment at the start of the workday and between patients), social distancing, compulsory masking for everyone, and stringent protocols for suspected COVID-19 patients. HCWs were acutely aware that using personal protective equipment (PPE) (not routinely worn pre-COVID-19) was critical. Closure of the entire clinic or certain services for fumigation was a frequent occurrence.

##### Service availability

HCWs reported that most essential services continued to be available with occasional inaccessibility due to extenuating circumstances such as staff shortages or closure for fumigation. Complete service closures during hard lockdown included circumcisions, pap smears and dental care. Initially, terminations of pregnancy were halted, but later reinstated since*, “There were a lot of patients who fell pregnant during lockdown”* (JHB HCW), and the procedure was time-sensitive. In hospital, elective procedures stopped. Outreach from hospitals to primary healthcare (PHC) facilities (e.g., allied health services) as well as from PHC to communities (e.g., tracing of defaulters and support groups) decreased or discontinued. This received mixed reviews from the HCWs, for “*With regards to the chronic patients, we have more defaulters… this was good and a bad thing, because some people were controlled just on lifestyle modification, and others we obviously had to re counsel and then restart medication”* (JHB HCW).

##### Platforms of care

CMs mostly reported using primary care facilities (clinics and community health centers), although some did access hospital services. Using private care was limited to the need for antenatal care or acute concerns such as suspected COVID-19 infection. A JHB CM expressed disappointment at how government clinics functioned and having to avail alternate facilities: “*…then you go back maybe after two days then they give you your treatment or they will not give you and tell you to go to another clinic then you get your treatment at that clinic or tell to go to Dischem pharmacy or private GP.”* Neither CMs nor HCWs reported using traditional healers, although the use of home remedies was widely discussed across both settings:… “*we were also using that home remedy of garlic and warm water to protect ourselves instead of going to the clinic or hospital to expose ourselves,”* (JHB CM); “*She* [mother] *had a sore throat, but she just uses traditional herbs, she used ginger and lemon*”(AGIN HCW).

##### Impact of service delivery changes

From the CM perspective, the impact of the COVID-19 related changes, was impeded access to services with exposure to harsh weather conditions, variable waiting times and uncertainty of assistance, especially for those with limited finances or who worked weekdays. Whilst CMs understood the need for these changes stating that HCWs, “*don't trust anyone, they have to protect themselves”* (AGIN CM), some viewed the HCWs being inefficient and, “*dragging their feet”* (JHB CM). Others regarded the changes as having improved the efficiency of care. Several CMs saw no difference in service delivery, explaining, “*Nothing much has changed. The difference is that before we used to queue inside the clinic and now, we queue outside the clinic”* (AGIN CM).

The impact of these changes on HCWs was related mostly to the increased burden of patient care. HCWs, like CMs, also had differing opinions on whether service changes improved or hindered efficient delivery of care. The latter was attributed to additional steps associated with tasks (e.g., donning of PPE) “*So, the first priority even if the patient is saturating, you have to take care of yourself, wear the protective gear before you jump and start resuscitating the patient. I would say in terms of service delivery, we are taking care of ourselves first before we were putting ourselves in harm's way”* (JHB HCW), and the increased time taken to complete certain tasks. For example, separating suspected COVID-19 patients meant that a chronic care patient with COVID-19 symptoms would require a HCW to fetch colleagues from the chronic section to consult with the patient in the COVID-19 tent. Some HCWs perceived a decreased patient load, especially during hard lockdown when patients were afraid to consult, unaware of service availability, or due to changes in consultation processes. A JHB HCW expressed, “*I think with the main clinic, there has been improvement. You would find that it would be packed all the way until 4 p.m. and they had to turn people away and tell them to come back the next day. But since COVID started, and with the chest clinic, because they see everyone with shortness of breath, sore throat, tonsils, things have changed. The workload has halved because it is now two different clinics.”*

However, most HCWs felt that the patient load was high, stating, “*for me it was difficult because during lockdown people were relaxed at their homes… But I was at work full time, no Saturdays, no holidays, no weekends. I was at work.”* (AGIN HCW). This was due to the combination of clinic and service closures, staff changes and movements (e.g., helping at other facilities), increased scope (e.g., COVID-19 screening and testing) and pressure to reach COVID-19 screening targets whilst still maintaining routine targets and Ideal Clinic Standards for essential services. Furthermore, HCWs with a broader skill set (such as doctors and professional nurses) observed that they bore the brunt of consulting COVID-19 patients was placed on them due to perceptions that these HCWs were more comfortable consulting with high-risk patients.

Ultimately HCWs reported feeling, “*strained and depressed because of the amount of work they had to do daily,”* (AGIN HCW), and felt like they were failing their patients, especially when their efforts went unrecognized. “*We are always overloaded with patients and some of the patients they even write about us on Facebook saying that we do not want to work we are very slow, but we are trying our best sometimes I don't even take my lunch time because I want to help them,”* an AGIN HCW recalled.

#### Service utilization

CMs reported usage of both acute and chronic care, despite COVID-19 related fears and financial constraints delaying health seeking for acute complaints. Recurrent concerns of both HCWs and CMs were related to fears of contracting COVID-19 at a facility, being alone in hospital, and dying in hospital. HCWs were suspicious that patients afraid of being reprimanded, lied about why they missed appointments and in the FGDs explained that while fear of contracting COVID-19 was a common excuse in their communities for not accessing care, there may have been other reasons.

HCWs' fear of contracting COVID-19 also influenced the experience of service utilization as CMs sensed that nurses were, “*afraid to touch us”* (AGIN CM). Conversely CMs' fear of contracting COVID-19 from HCWs meant that home visits were not welcomed.

With regards to performing healthcare procedures in a COVID-19 tent, HCW indicated that some patients felt stigmatized when triaged there or that care they received was suboptimal. Other HCWs however, viewed it as an easy and efficient way to be seen to without entering a facility: “*Also, I think it's [efficient] because of the COVID triage. So, at the front of the clinic they are doing a triage, where if you have a cough; if you have all those COVID symptoms, you are treated there at the tent. A lot of the symptoms, like flu, are not being seen here.”* (JHB HCW)

HCWs at both clinics and hospitals noticed a decrease in ill children, and in trauma cases, which they attributed to the inaccessibility of alcohol, closed schools and decreased mobility. HWCs noted that during hard lockdown only severely ill patients presented to facilities. As lockdown eased, district hospitals experienced an influx of patients, particularly with chronic conditions, seeking care.

“*The actual fact that alcohol stopped at some point, and our trauma decreased significantly. We were focusing our time on really serious cases that were not so much trauma related and that was lovely. But the hardest part of the lockdown was that COVID cases went up even though the trauma decreased, the COVID and respiratory distresses started picking up drastically.” (*AGIN HCW)

#### Information and communication

According to HCWs, the inability to hold meetings regarding service or process changes, meant that communication among HCWs and between HCWs and CMs relied on word of mouth through facility personnel and community health workers (CHW)s. Using social media addressed communication gaps amongst HCWs themselves without contravening social distancing protocols where, “*WhatsApp groups had to come into play … videos were being shared … So, I would say they did attempt to train us virtually, but it wasn't as smooth as it would have been had it been the normal way,”* (JHB HCW). Despite the attempts some HCWs inferred that communication on the correct COVID-19 treatment protocols was still lacking, “*You would be gathered for management to tell you that, ‘we have changed protocol'. Changing it from which one to start with? And only then would you realize that there was a protocol. Then, the next thing is that they change it again,”* explained an AGIN HCW. Other strategies included notices posted on clinic gates.

Although in some areas, HCWs living in the community they served and staff within the facility described substantial amounts of time being spent relaying updated service information, CMs reported that they usually were only aware of changes upon reaching the facility or through other CMs. A CM from Agincourt stated, “*We are confused, we do not know whether it is because of corona or what, we do not understand because they don't explain anything.”*

Individual patient-based communication like medication and appointment reminders were done *via* telephonic follow up and short messaging systems (SMS) for Central Chronic Medicines Dispensing and Distribution (CCMDD) patients (24). However, when interventions included having to employ computers and technology, HCWs expressed feeling overwhelmed. An AGIN HCW explained, “*If they can hire someone who can work with CCMDD that will be much better because I am nurse, but I do not know how to use a computer, it needs someone who knows how to use a computer.”*

Opportunistic waiting room health education was stopped due to the limited numbers of patients allowed within the facility. This necessitated health promotion activities being communicated through door-to-door campaigns and *via* community radio. CMs reported that health education activities addressed COVID-19 at the expense of other conditions: “*The government is looking at one side and that side is focusing on COVID and forgetting that there are people living with other conditions and taking treatment*” (JHB CM).

#### Health workforce

Staffing levels at facilities were impacted due to staff illnesses, deaths, and protection of high-risk individuals, as well as the “*50/50”* arrangement (staff rotation where the complement is 50% per day) to mitigate staff exposure to COVID-19. COVID-19 exposure protocols demanded immediate quarantining of all exposed staff whilst waiting for often delayed test results. This meant that, “*if someone goes to quarantine for a very long time, you are short (staffed), if there are 3 of you, you will hold this clinic together for those 10 days without people.”* (JHB HCW). This scenario, however, was not always practiced, where to avoid being short-staffed, HCWs who suspected they had contracted COVID-19 often continued working while waiting for their test results, potentially exposing co-workers to infection. A JHB HCW revealed, “*You work while you wait for your results then when they are positive that is where you get worried.”*

This staff shortage was partially addressed by hiring contract staff, using private general practitioners and NGO staff. However, HCWs maintained that these measures were not enough to address the workload. Reallocation of roles was prevalent, with a AGIN HCW stating that there was a “*team spirit”* with everyone working collaboratively to meet service delivery needs. This sentiment was also raised by CMs, who were more cognizant of new staff at the facility than staff shortages. They perceived new staff to be friendlier and the attitudes at the clinic to have positively changed, “*because the old staff is no longer there, the nurses.”* (JHB CM)

CHW roles were impacted most. They reported a range of additional duties including COVID-19 community screening and testing, medication delivery, screening at facilities and administrative duties, whilst their usual community-based activities were “*left behind”* (AGIN HCW). Health promoters stopped their school-based activities due to school closures, assisting instead in communities and facilities as needed. Nurses, clinical associates, and doctors reported spending more time than usual on management duties, and doctors were tasked with occupational health duties such as reviewing ill colleagues and completing necessary documentation for occupational disease compensation.

HCWs felt that they were inadequately trained to relay COVID-19 advice to patients, especially when standard information did not consider patients' circumstances, e.g., lack of adequate water to wash hands. A JHB HCW reiterated, “*With us healthcare workers, we are pleading with them to take us for training/ in service training on time. It should not be a matter of rush… Then they put us in things [new tasks and roles], and we are also not ready with those things”* (JHB HCW). HCWs, like one from JHB clarified that although they did not always have the necessary skills, “Y*ou had to be a Jack of all trades”* to fulfill the new roles. Some doctors further lamented unrealistic expectations placed on them by non-doctor HCWs: “*Because you are a doctor it is assumed that you are able to do anything that they cannot do.”* The lack of training and uncertainty around the disease protocols caused anxiety among all categories of healthcare staff. Nonetheless, some HCWs, especially CHWs, appreciated their new roles and respected the associated responsibilities. They explained “*when you are a community health worker you are the source of information for them before even this pandemic happened and it you give them the right information when they need it, they will always value you, including pandemics like the COVID-19”*.

Managers were often seen as unsupportive and lacking empathy. This was due to the absence of debriefing, unwillingness to entertain and address complaints, and lack of concern for ill staff. HCWs were distressed that their safety was disregarded despite safety being a requirement to continue service delivery. They felt that they were sometimes not informed of situations where they could have been exposed to COVID-19. This included situations where CHWs had to screen positive households contacts, or instances where safety protocols were breached, e.g., inconsistent criteria for clinic closure for fumigation or lack of PPE. The actions of management resulted in anger and frustration. Some HCWs participated in strikes and report having to fight for their needs to be addressed: “*And it was a huge thing we had to fight with the matron (facility manager), because I was not prepared to work without a PPE.”* (JHB HCW) This sense of agency and force was reported more by doctors, clinical associates, and professional nurses than health promoters and CHWs.

#### Medication and resources

According to HCWs and CMs, the supply of medicines was like that prior to COVID-19. A CM stated that when stock-outs occurred they were, “*not disappointed because [the community] know that is how the clinic is.”* (JHB FGD). While one HCW reflected that, “*somebody somewhere wasn't doing his job because… since COVID everything is here, especially those cough medications, those are not lacking at all”* (JHB HCW), others were concerned about increased stock-outs especially of acute medication such injectables for immunization, and consumables such as glucose testing strips. Certain medications (e.g., Vitamin C) were impacted since their use was prioritized for suspected COVID-19 patients. Other reasons HCWs suggested for stock outs included an influx of patients from other provinces or facilities. In response to the unanticipated increase in demand, HCWs “shared” the medication between patients, so patients received less than required. HCWs also improvised by borrowing medications from other facilities or prescribing acute medications for purchase at private pharmacies.

For chronic medications, HCWs and CMs described an increase in pre-packaging practices, automated dispensing machines, usage of private pharmacies as external pick-up points, SMS notification of medication being ready for collection, dispensing of medication for longer periods and home delivery of medication through CHWs, to decrease facility visits and improve access. This was well received as noted by a CM in Agincourt: “*Now it is simple for us when I go and collect my medication…. I don't have any difficulties because now they send us SMS to collect our medication at [the] pharmacy*.”

CMs who felt at high risk of contracting COVID-19 or were otherwise unable to attend facilities, reported sending loved ones to collect their medication. In certain instances they also reported being able to access medication from facilities at which they were not usually a patient.

“*When COVID started and we couldn't travel on my date of return to the clinic, I just went to the clinic there in Phomolong and they helped me. They gave me the same medication. They did ask me if I was transferred and why, but I told them it was due to the pandemic. I gave them my boxes for my medication, and they gave me the same.”* (JHB CM)

These practices were viewed positively by CMs who perceived them as improving both efficiency and access, especially for those unable to take time off work. Those who already had participated in programs like CCMDD perceived that these processes were even more efficient during COVID-19.

#### Financial impact

Although differing in severity, both HCWs and CMs relayed financial impacts related to healthcare service delivery. For HCWs this included income changes due to lack of annual salary increases and salary cuts resulting from changes in working hours and working overtime without pay. Some suffered a loss of increments related to additional qualifications, since systems on which to upgrade qualifications were inactive during COVID-19. However, the formalization of some staff from contract to permanent employment meant a steadier income.

Additionally, some HCWs incurred unexpected costs resulting from PPE expenses as “*the government was not able to give us something to protect us”* (AGIN HCW), as well as essential equipment such as portable oxygen saturation machines (for use in the absence of electricity). Despite free COVID-19 testing being available, exposed HCWs sometimes paid to test privately due to the faster turnaround time. They also incurred data costs to access patient results on their mobile phones. For CMs, service delivery changes (mainly around triaging and medication availability), and poor communication caused them to have increased expenses for transport or to access private service providers for consultations and medication. This added to the existing financial constraints brought on by loss of employment and/or income.

#### Quality of care

HCWs noted many ways in which the quality of care they delivered was impacted. The high patient load and fragmentation of services into COVID-19 and non-COVID-19, meant that holistic patient care was compromised, with a JHB HCW stating that, “*it didn't allow us to manage patients holistically because if now the workload is too much you wouldn't do everything because now you are having 100 patients waiting for you, so you end up doing half the job. We would just issue medication and the patient leaves… whatever else that could be wrong was not the concern.”* This shortcoming was noted by CMs also, who felt at times that not all their needs were addressed, claiming*, “the doctors were also afraid to speak to us, to interact with us, to check us, they only just gave us medication,” (*JHB CM).

HCWs felt pressured to work even faster, spending “*not more than 3 min”* (JHB HCW) with patients, to manage the patient load and match the standard of service delivery prior to COVID-19,” with some claiming, “*sometimes in the end there were a lot of mistakes that were happening,”* (AGIN HCW).

HCWs and CMs reflected on the strained relations between patient and clinician resulting from measures like shorter consultation times, social distancing and mask wearing, particularly when sensitive matters required discussion. “*So, before the pandemic, when you're dealing with a patient inside the consultation room, the patient used to be able to be very close to you, but now the patient must move 1 to 2 meters away from you. Now, think about it, the patient is trying to tell you the problem, a very private one but now they can't get close to you. So it did compromise the relationship between us and the patients.”* (JHB HCW)

The lack of physical contact during the consultation process was of concern to both CMs and HCWs. HCWs were frustrated because “*you had to learn mostly how to examine a patient with minimal contact, minimal touching” (*JHB HCW). Whilst CMs commented on and empathized with the HCWs' fear of touching patients, HCWs were equally aware that the lack of contact meant that patients would feel as if their problems were not addressed. “*Patients feel that if you do not touch them, you didn't do anything to them. So, at the end of the day they still go home feeling like they're still sick because you didn't touch their stomach. So, now you need to be able to ask specific questions to understand whether the pain is in the chest or in the stomach, and I am supposed to touch you for this but now according to the rules, I can't.”* (JHB HCW)

#### Recommendations for strengthening the health system beyond COVID-19

HCWs and CMs believed the health systems emergency preparedness was lacking. Furthermore, the current state of health services needed to be improved to ensure that service delivery disruptions would be minimized when public health emergencies arose. This included improvements to infrastructure, increasing human resources and improving resource availability for health service delivery. Additionally, robust communication systems and processes for patient education was seen as important. The arrangements to decentralize care *via* home visits, delivery of medication and mobile clinics as well as protocols for extended dispensation of medication for stable patients were seen as initiatives that would have improve service delivery, going forward.

Other systems that HCWs suggested needed to be in place to optimize healthcare and service delivery, included an increased focus on prevention of disease and promotion of health as well as surveillance systems (similar to those of TB and HIV) to track chronic diseases.

Whilst other recommendations from CMs centered on social circumstances such as ensuring job security and access to food, those from HCWs centered around initiatives to support HCWs in the workplace such as emergency preparedness training for HCWs; psychological support, empathy, and debriefing; and including front-liners in service delivery decisions. Nevertheless, HCWs and CMs believed that the government and health services did the best they could under the circumstances but there would always be room for improvement. “*I think the government did pretty well. We are very entitled, and we are harsh on them. … I think they did an okay job. Nothing is ever going to be perfect. Yes, we need to improve on what we are currently doing”* (JHB HCW).

## Discussion

In this study HCWs and CWs were interviewed to glean their perceptions of the quality of healthcare and service delivery that they experienced at the onset of South Africa's national lockdown from March 2020 to September 2021, to ascertain the impact of the more intense restrictions pertaining to movement and service access during this period.

The results in this paper are reflective of the evolving nature of the pandemic. There were varying experiences of service delivery, resulting from the availability of new information, best practices, and governments' responses to these. This is evidenced in the WHO Continuity Surveys' report which states that 27% of countries experienced disruption to 75–100% of their services in 2020. This changed to 9 and 18% of countries in 2021 and 2022, respectively, still experiencing interruptions 2 years later (25–27).

Globally NCD services experienced more disruptions than MCH and communicable disease services. Communicable diseases has been more resilient due to years of global investment into HIV and tuberculosis (9, 28, 29). The impact on NCDs in countries similar to South Africa arose from staffing shortages and reallocations to the COVID-19 response as well as decreases in utilization (30). This is globally concerning, given the increased risk of COVID-19 and death for those with NCDs, and the long-term impact of COVID-19 interventions. The consequences of NCD treatment interruptions and delayed health seeking are already evident in some countries where hospital admissions for uncontrolled, severe chronic conditions have spiked (30–33).

MCH faring better than NCD treatment proved to be partially true in in our study. While MCH patients were attended to first, utilization in MCH still dropped. Other studies indicate that despite efforts to continue service availability, MCH services utilization was hampered (25–27, 34–36). Given that routine immunization was compromised according to our study participants, it raises additional concerns as modeling studies show decreases in immunization coverage of at least 7% for measles-containing vaccines, with associated outbreaks in many countries (35, 37–39). Furthermore, HCWs' opinion that the COVID-19 pandemic may have resulted in increased pregnancies and terminations echoed that of other studies which concluded that that the COVID-19 pandemic may have resulted in increased adolescent and unintended pregnancies, maternal deaths, and stillbirth rates (40–42).

With respect to health service adjustments to overcome the challenges faced, South Africa, like other countries (25–27), implemented strategies to decrease patient loads and respond to needs by changing consultation routines and increasing staff capacity. Despite this, HCWs reported still experiencing high volume workloads (which would have further been exacerbated once the COVID-19 vaccination rollouts commenced). Unlike many other countries (27) where interventions like telemedicine and home based care were prevalent, in South Africa, telemedicine was limited to managing chronic patients *via* telephonic reminders to collect medication and keep appointments. In addition, CHWs, who have been recognized as a critical health system resource during pandemic and non-pandemic times (43), were utilized mainly for COVID-19 related activities and were frustrated that their usual home based activities were hampered and in instances, stopped altogether (44).

Whilst 43% of countries reported disruptions in prescription renewals for chronic medications and challenges with essential medicine (27, 30), our study participants did not perceive much of a difference in medicine availability when compared to pre-COVID-19. The CCMDD program, initiated in 2014, saw rapid scale-up for chronic patients (45–48) and was well-received by both HCWs and CMs due to increases in efficiency and convenience and decreases in patient load and financial impact on patients (46).

Nevertheless, service delivery and utilization were negatively impacted by the fear of contracting COVID-19 as expressed by HCWs and CMs. A cycle of blame ensued, where HCWs felt that CMs were negligent in reporting COVID-19 symptoms or seeking treatment and CMs felt that HCW were providing them with sub-standard care. Avoidance behavior resulted from each group's fear of contracting the disease from the other. Inevitably, the quality of care offered by HCW was compromised (20, 43).

SA is amongst many health systems that have experienced setbacks in both health service delivery and momentum toward UHC (49). Participants in this study noted that whilst the SA government did their best under the circumstances, pre-existing gaps and inadequate resilience in the health system contributed to service delivery challenges.

Whilst strengthening the individual health system building blocks and public health response measures are necessary to fortify resilience in the face of the ongoing and further pandemics (50) as well as progress toward UHC in SA (49), three areas that require increased efforts in SA are: PHC based health system strengthening with a strong community focus to encourage a collaborative approach (51) toward creating solutions around the issue of health-care; usage of e-/mHealth platforms to optimize service delivery (52) particularly during times when patient movement between residence and health care facilities should be minimized; and overall investment in priority setting mechanisms (53) to be able to plan for and effect mitigation strategies equitably during possible future communicable disease pandemics.

PHC systems such as home-based care including prevention and promotion activities, home delivery and decentralized medication systems are critical to improving access to care and efficiency of services whilst ensuring that ill patients can be seen to timeously at the relevant facility. These interventions can be further augmented by mechanisms to monitor stable patients remotely through digital health interventions. However, consideration would need to be given to the fact that whilst some application of telehealth is available in South Africa, widespread use has been hampered by lack of national policy (54) due to the anticipated costliness of initializing and then sustaining telehealth programs. In addition, the Health Professions Council of SA regulations for clinicians (55) which advises that telemedicine functions best in situations where a patient-practitioner relationship already exists, and that such services may be charged for, have further delayed its adoption since rules governing the execution of telemedicine have yet to be formalized. Thus, more work is needed to understand how telemedicine models in SA and globally could be adapted for widespread use in the public sector and the infrastructure required, so that these service lines achieve their intended outcomes and do not further impede access, quality, efficiency or equity (54, 56).

Finally, priority setting mechanisms are a key aspect of health system functioning that allow decision makers to deliver much needed services during stability and to pivot easily with minimal impact during public health emergencies. The WHO continuity survey highlights that 25 countries expressed technical assistance needs with respect to service package priority setting and resource allocation (27). With the further erosion of public trust in health systems during COVID-19, transparent and consistent priority setting mechanisms are needed to both improve the trust needed to combat the ongoing pandemic as well as to allow for rebound of health systems as they attempt to regain the losses to UHC attainment (57–61).

### Strengths and limitations

The strength of this study is that it provides perceptions and lived experiences of both CMs (recipients of healthcare) and HCWs (providers of healthcare) from both South African urban and rural contexts.

Recruiting male participants into the research was challenging. Under-recruitment of them and loss to follow up among those men who were recruited resulted in a gender imbalance in our sample, particularly for FGDs where participants were only women.

It is possible then, that had an equal number of men and women been surveyed, other patterns of responses based on men's perceptions vs. women's perceptions, may have emerged. There is a need to identify and employ an alternative strategy to recruit men in future research.

The interview process itself was hampered due to COVID-19 regulations as in-person interviews in Johannesburg often had to be replaced by telephonic interviews. The quality of some telephonic recordings was impacted by cellular reception, electricity failures and background disturbances. Furthermore, while respondents were reminded to reflect on their experiences across the whole period of multiple lockdown levels, recall bias may possibly have influenced the experiences reported by them, 12–18 months after the first hard lockdown.

## Conclusion

Concessions and novel strategies to avail medication to patients had to be created. Since providence was lacking, government needs to formulate health intervention strategies that embrace health literacy, alternate methods of chronic medication dispensation, improved communication across health care platforms and the use of telehealth, to circumvent the threats of possible further infectious disease outbreaks.

## Data availability statement

The raw data supporting the conclusions of this article will be made available by the authors, without undue reservation.

## Ethics statement

The studies involving human participants were reviewed and approved by the University of the Witwatersrand Human Research Ethics Committee (M201084) in January 2021. Departmental approval was granted in February 2021 by the Johannesburg Health District (DRC Ref: 2020-11-015), Mpumalanga Province Ethics Committee (MP_202102_03), and National Health Research Database (GP_202011_066). Community members received ZAR 300 (USD 20.36) reimbursement for their time and travel in Johannesburg, whereas interviews were conducted at respondent's home in the Agincourt HDSS. The patients/participants provided their written informed consent to participate in this study.

## Author contributions

Conception: KH and SG. Data collection: AN, SN, and JH. Data analysis and interpretation: AE, AM, JH, JP, SG, SL-E, and TR-P. Drafting manuscript: AE, AM, JH, and SL-E. Critical revision: AM and SL-E. Final approval of the submitted versions: AE, AN, AM, JH, JP, KH, KK, SG, SN, ST, SL-E, and TR-P. All authors contributed to the article and approved the submitted version.
